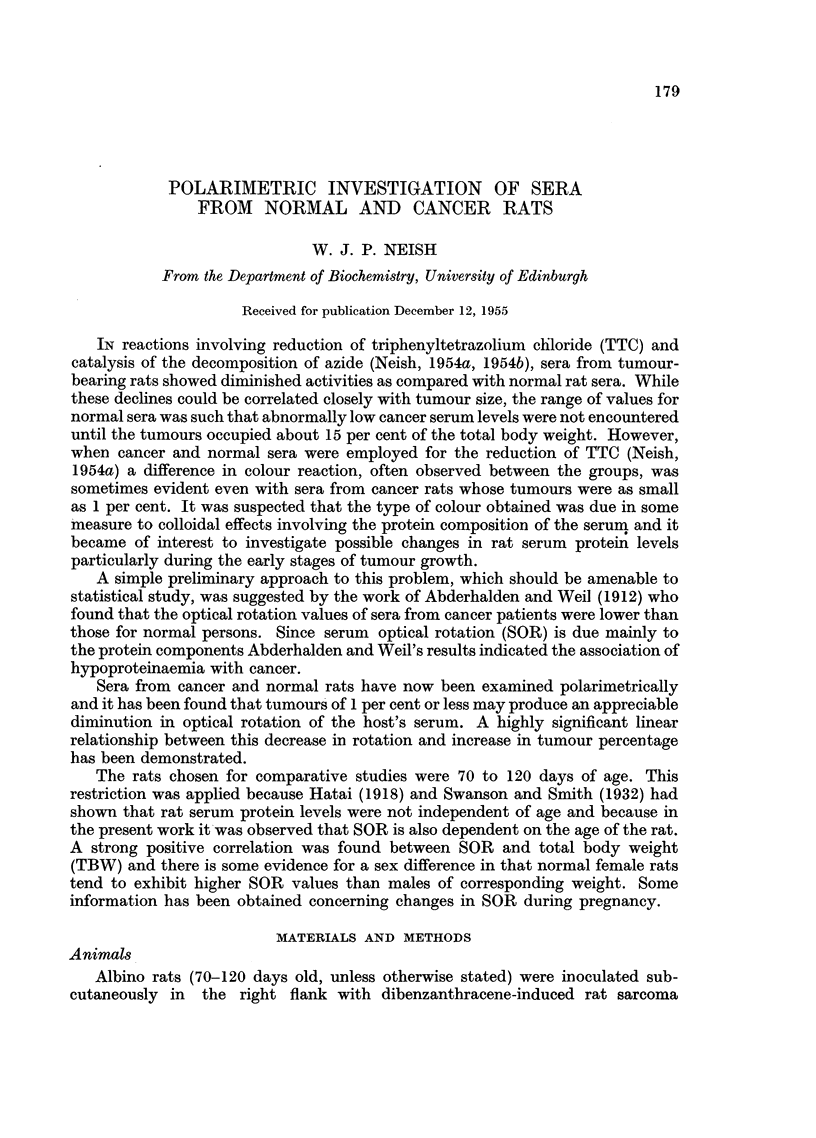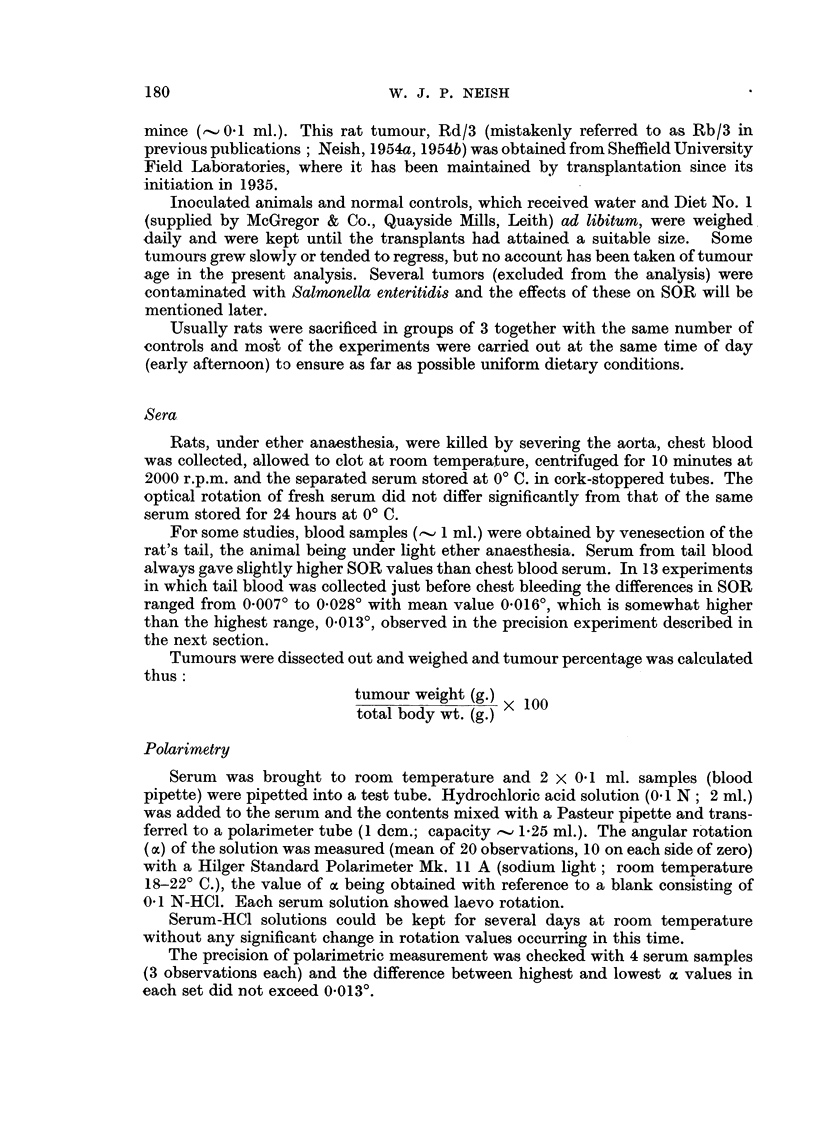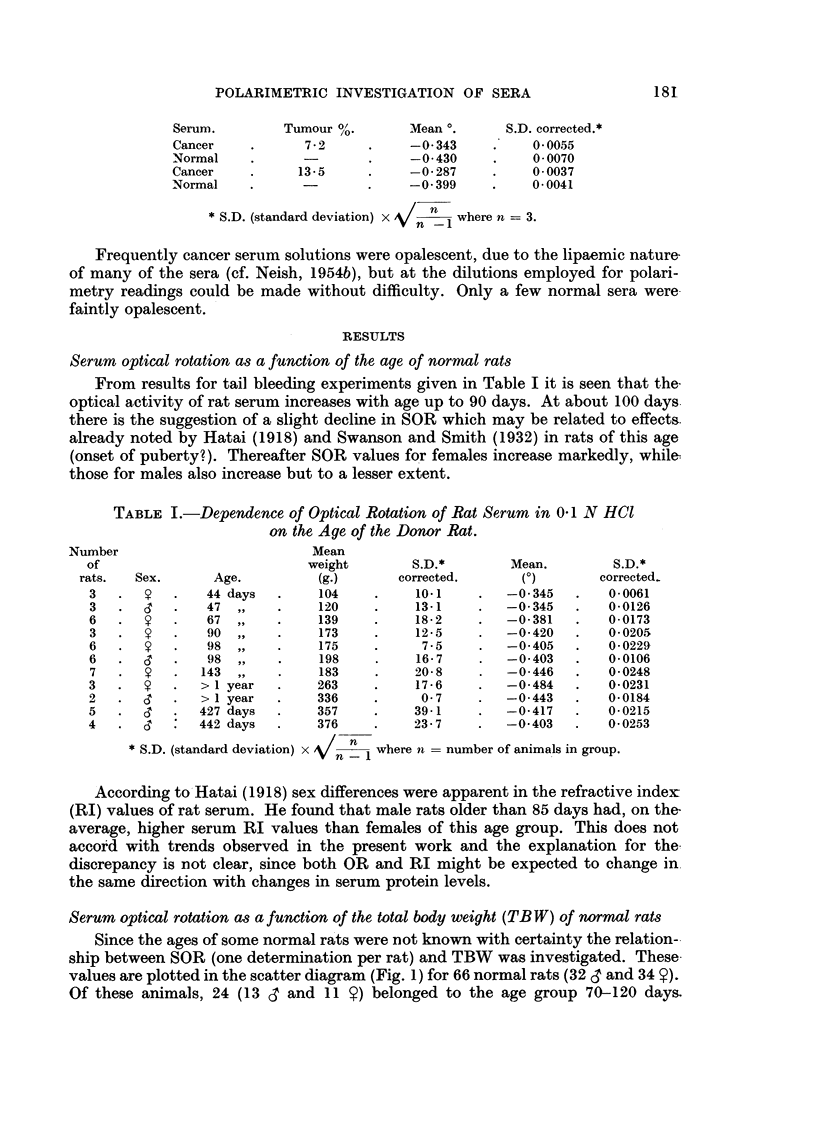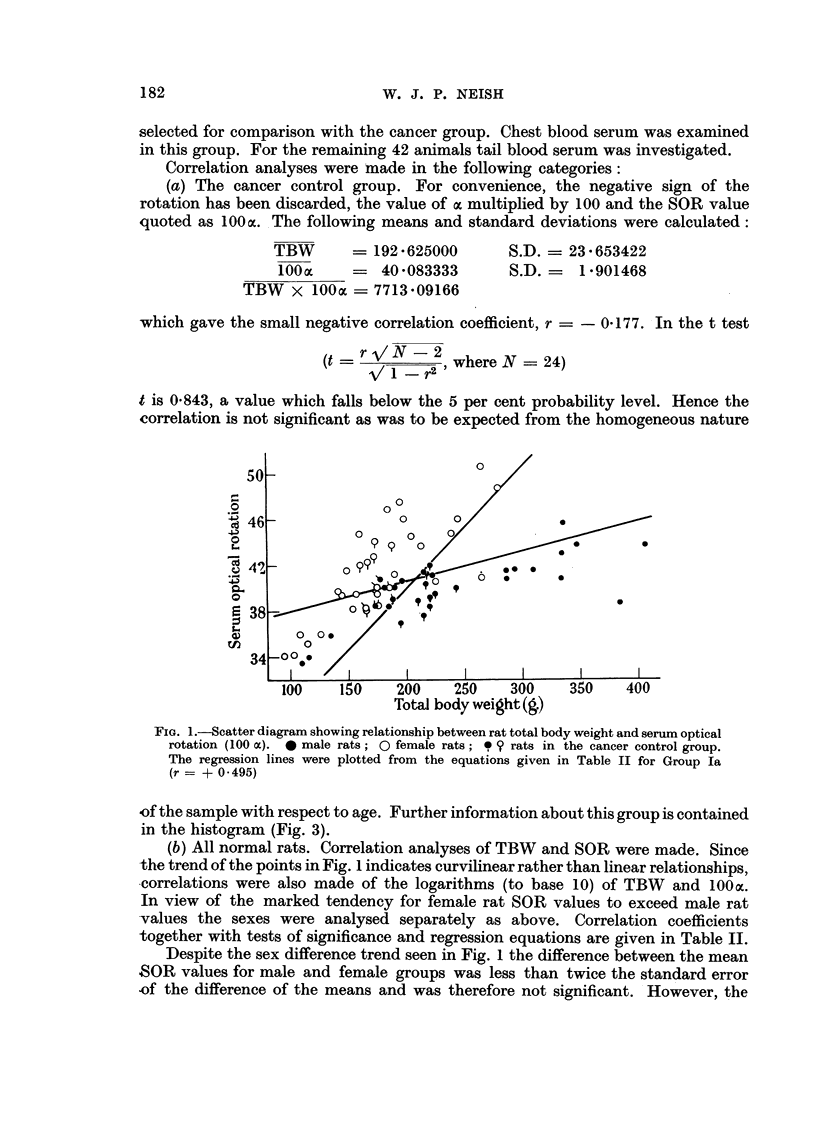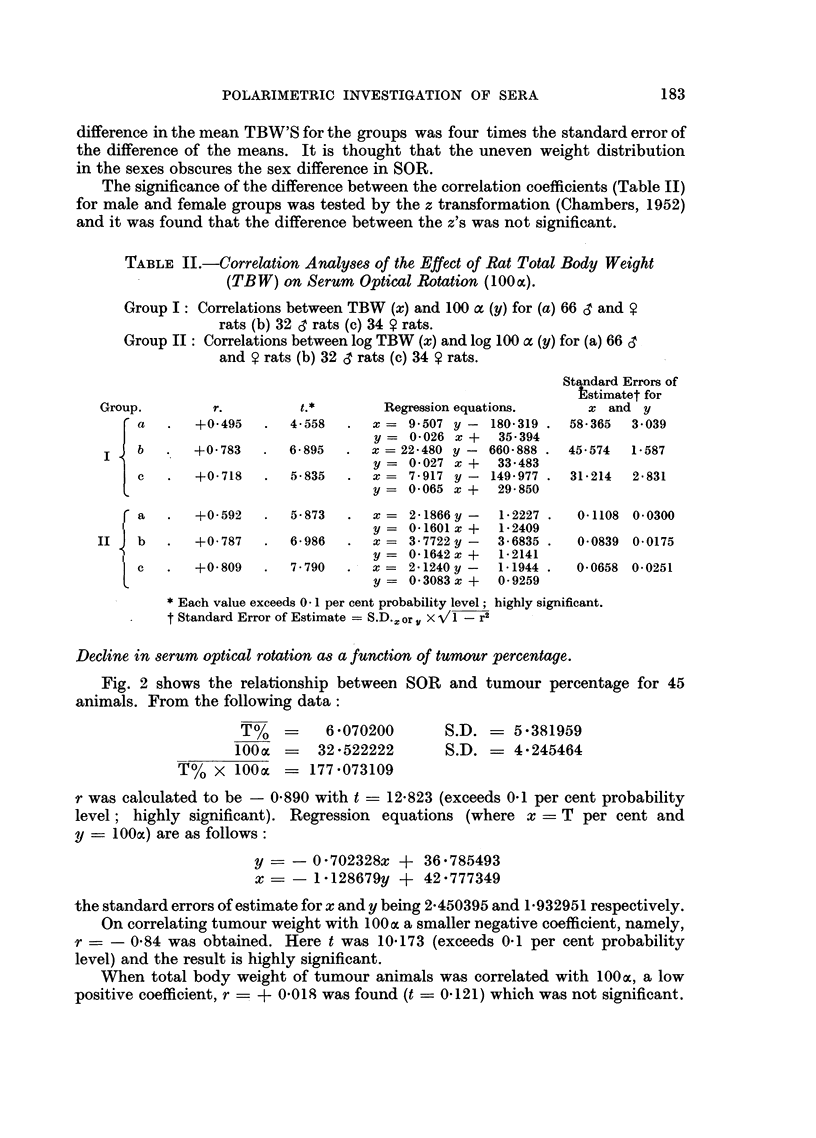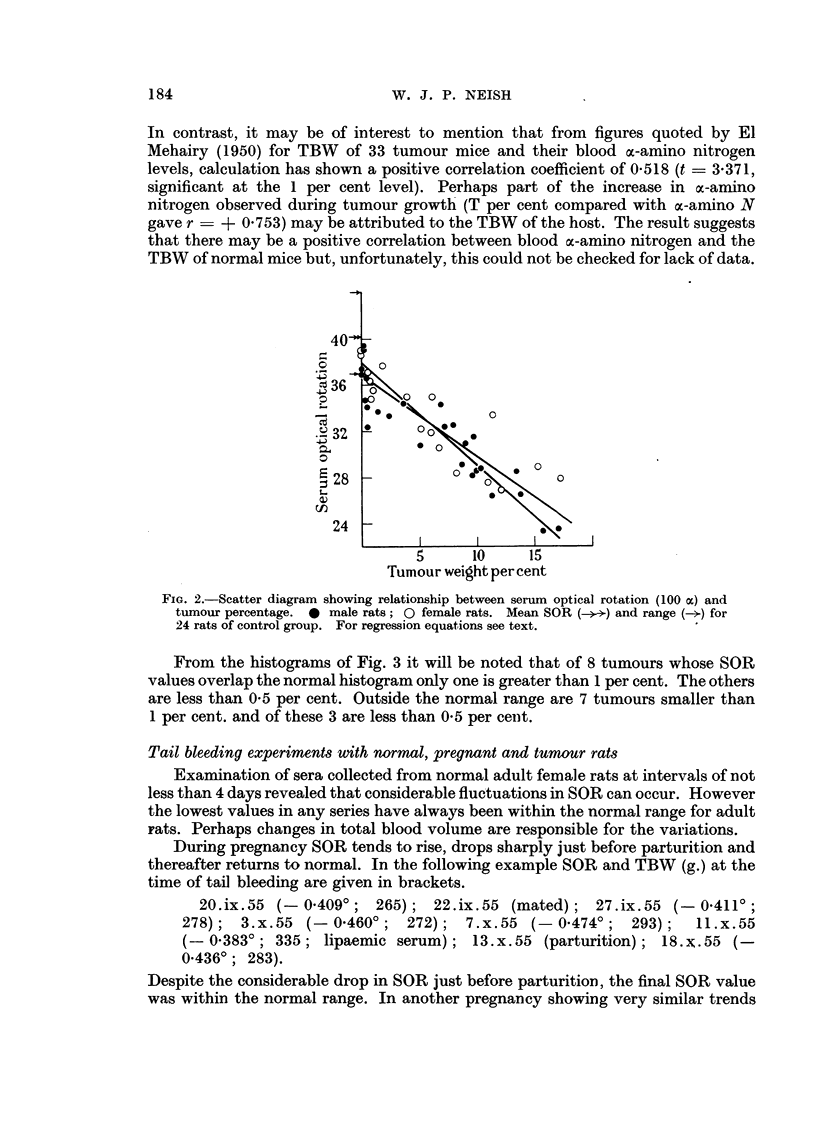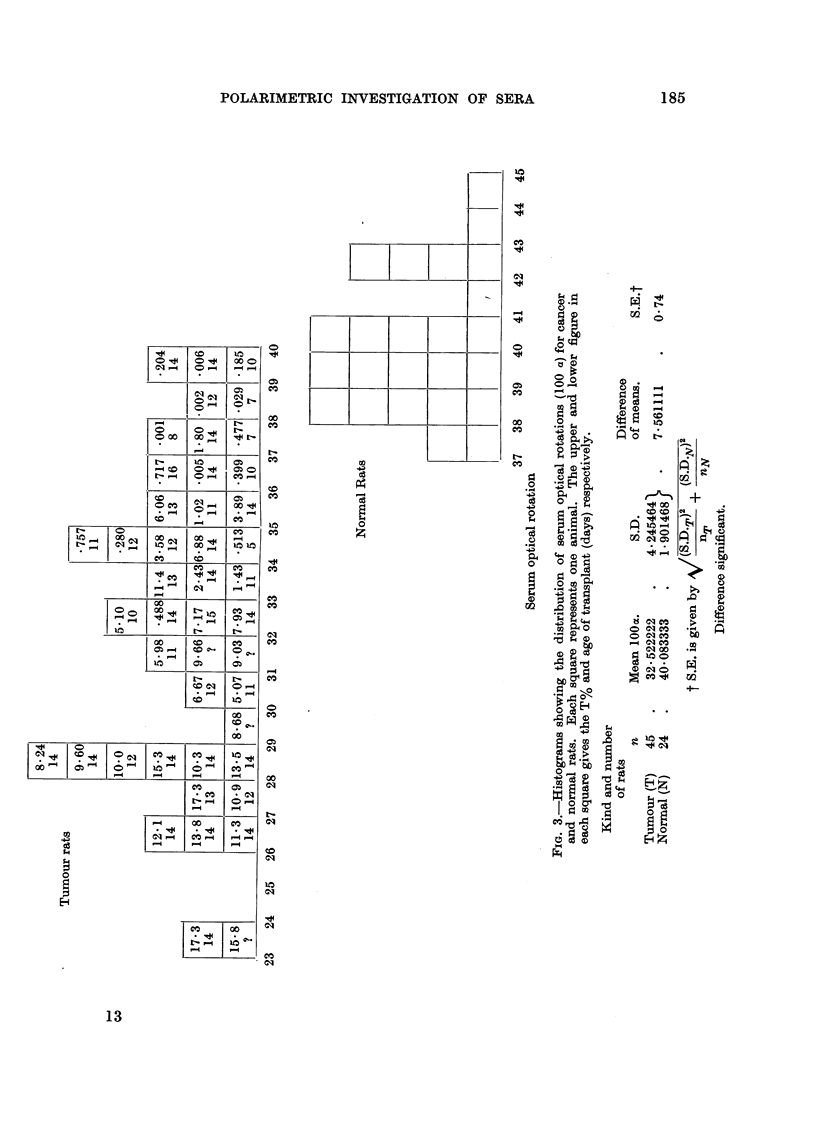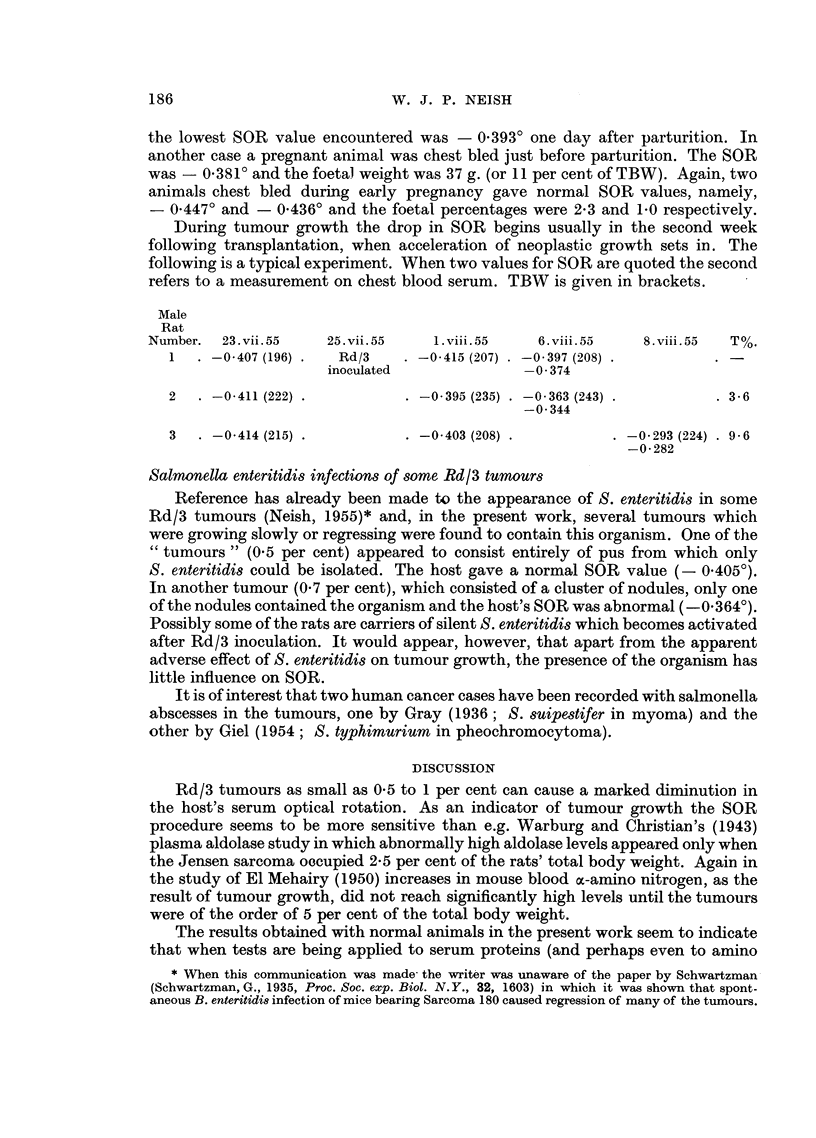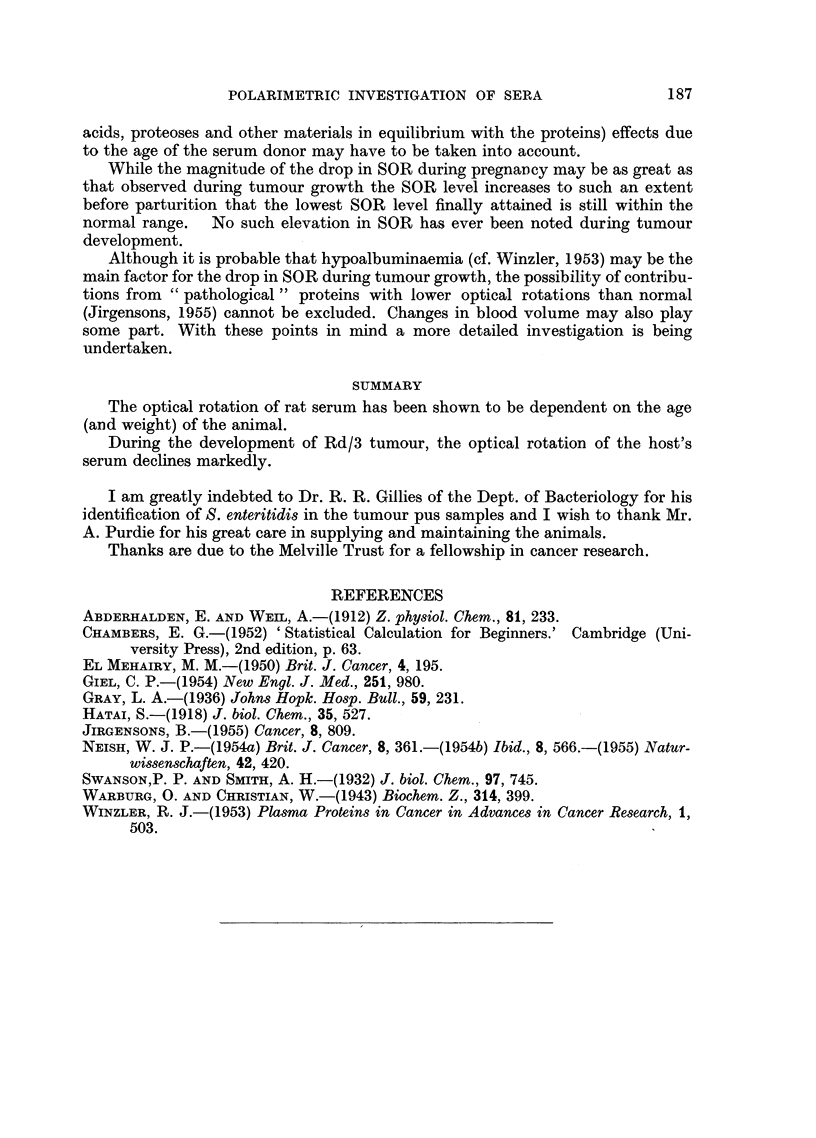# Polarimetric Investigation of Sera from Normal and Cancer Rats

**DOI:** 10.1038/bjc.1956.21

**Published:** 1956-03

**Authors:** W. J. P. Neish


### POLARIMETRIC INVESTIGATION OF SERA FROM NORMAL AND CANCER RATS

W. J. P. NEISH

*From the Department of Biochemistry, University of Edinburgh*

Received for publication December 12, 1955

IN reactions involving reduction of triphenyltetrazolium chloride (TTC) and catalysis of the decomposition of azide (Neish, 1954*a*, 1954*b*), sera from tumour-bearing rats showed diminished activities as compared with normal rat sera. While these declines could be correlated closely with tumour size, the range of values for normal sera was such that abnormally low cancer serum levels were not encountered until the tumours occupied about 15 per cent of the total body weight. However, when cancer and normal sera were employed for the reduction of TTC (Neish, 1954*a*) a difference in colour reaction, often observed between the groups, was sometimes evident even with sera from cancer rats whose tumours were as small as 1 per cent. It was suspected that the type of colour obtained was due in some measure to colloidal effects involving the protein composition of the serum and it became of interest to investigate possible changes in rat serum protein levels particularly during the early stages of tumour growth.

A simple preliminary approach to this problem, which should be amenable to statistical study, was suggested by the work of Abderhalden and Weil (1912) who found that the optical rotation values of sera from cancer patients were lower than those for normal persons. Since serum optical rotation (SOR) is due mainly to the protein components Abderhalden and Weil's results indicated the association of hypoproteinaemia with cancer.

Sera from cancer and normal rats have now been examined polarimetrically and it has been found that tumours of 1 per cent or less may produce an appreciable diminution in optical rotation of the host's serum. A highly significant linear relationship between this decrease in rotation and increase in tumour percentage has been demonstrated.

The rats chosen for comparative studies were 70 to 120 days of age. This restriction was applied because Hatai (1918) and Swanson and Smith (1932) had shown that rat serum protein levels were not independent of age and because in the present work it was observed that SOR is also dependent on the age of the rat. A strong positive correlation was found between SOR and total body weight (TBW) and there is some evidence for a sex difference in that normal female rats tend to exhibit higher SOR values than males of corresponding weight. Some information has been obtained concerning changes in SOR during pregnancy.

### MATERIALS AND METHODS

### *Animals*

Albino rats (70–120 days old, unless otherwise stated) were inoculated subcutaneously in the right flank with dibenzanthracene-induced rat sarcoma mince ($\sim$ 0·1 ml.). This rat tumour, Rd/3 (mistakenly referred to as Rb/3 in previous publications ; Neish, 1954*a*, 1954*b*) was obtained from Sheffield University Field Laboratories, where it has been maintained by transplantation since its initiation in 1935.

Inoculated animals and normal controls, which received water and Diet No. 1 (supplied by McGregor & Co., Quayside Mills, Leith) *ad libitum*, were weighed daily and were kept until the transplants had attained a suitable size. Some tumours grew slowly or tended to regress, but no account has been taken of tumour age in the present analysis. Several tumors (excluded from the analysis) were contaminated with *Salmonella enteritidis* and the effects of these on SOR will be mentioned later.

Usually rats were sacrificed in groups of 3 together with the same number of controls and most of the experiments were carried out at the same time of day (early afternoon) to ensure as far as possible uniform dietary conditions.

### *Sera*

Rats, under ether anaesthesia, were killed by severing the aorta, chest blood was collected, allowed to clot at room temperature, centrifuged for 10 minutes at 2000 r.p.m. and the separated serum stored at 0° C. in cork-stoppered tubes. The optical rotation of fresh serum did not differ significantly from that of the same serum stored for 24 hours at 0° C.

For some studies, blood samples ($\sim$ 1 ml.) were obtained by venesection of the rat's tail, the animal being under light ether anaesthesia. Serum from tail blood always gave slightly higher SOR values than chest blood serum. In 13 experiments in which tail blood was collected just before chest bleeding the differences in SOR ranged from 0·007° to 0·028° with mean value 0·016°, which is somewhat higher than the highest range, 0·013°, observed in the precision experiment described in the next section.

Tumours were dissected out and weighed and tumour percentage was calculated thus :

$$\frac{\text{tumour weight (g.)}}{\text{total body wt. (g.)}} \times 100$$

### *Polarimetry*

Serum was brought to room temperature and 2 × 0·1 ml. samples (blood pipette) were pipetted into a test tube. Hydrochloric acid solution (0·1 N ; 2 ml.) was added to the serum and the contents mixed with a Pasteur pipette and transferred to a polarimeter tube (1 dcm.; capacity $\sim$ 1·25 ml.). The angular rotation ($\alpha$) of the solution was measured (mean of 20 observations, 10 on each side of zero) with a Hilger Standard Polarimeter Mk. 11 A (sodium light ; room temperature 18–22° C.), the value of $\alpha$ being obtained with reference to a blank consisting of 0·1 N-HCl. Each serum solution showed laevo rotation.

Serum-HCl solutions could be kept for several days at room temperature without any significant change in rotation values occurring in this time.

The precision of polarimetric measurement was checked with 4 serum samples (3 observations each) and the difference between highest and lowest $\alpha$ values in each set did not exceed 0·013°.

| Serum. | Tumour %. | Mean °. | S.D. corrected.* |
|---|---|---|---|
| Cancer | 7·2 | −0·343 | 0·0055 |
| Normal | — | −0·430 | 0·0070 |
| Cancer | 13·5 | −0·287 | 0·0037 |
| Normal | — | −0·399 | 0·0041 |

* S.D. (standard deviation) $\times \sqrt{\frac{n}{n-1}}$ where $n = 3$.

Frequently cancer serum solutions were opalescent, due to the lipaemic nature of many of the sera (cf. Neish, 1954*b*), but at the dilutions employed for polarimetry readings could be made without difficulty. Only a few normal sera were faintly opalescent.

### RESULTS

### *Serum optical rotation as a function of the age of normal rats*

From results for tail bleeding experiments given in Table I it is seen that the optical activity of rat serum increases with age up to 90 days. At about 100 days there is the suggestion of a slight decline in SOR which may be related to effects already noted by Hatai (1918) and Swanson and Smith (1932) in rats of this age (onset of puberty?). Thereafter SOR values for females increase markedly, while those for males also increase but to a lesser extent.

TABLE I.—*Dependence of Optical Rotation of Rat Serum in* 0·1 *N HCl on the Age of the Donor Rat.*

| Number of rats. | Sex. | Age. | Mean weight (g.) | S.D.* corrected. | Mean. (°) | S.D.* corrected. |
|---|---|---|---|---|---|---|
| 3 | ♀ | 44 days | 104 | 10·1 | −0·345 | 0·0061 |
| 3 | ♂ | 47 „ | 120 | 13·1 | −0·345 | 0·0126 |
| 6 | ♀ | 67 „ | 139 | 18·2 | −0·381 | 0·0173 |
| 3 | ♀ | 90 „ | 173 | 12·5 | −0·420 | 0·0205 |
| 6 | ♀ | 98 „ | 175 | 7·5 | −0·405 | 0·0229 |
| 6 | ♂ | 98 „ | 198 | 16·7 | −0·403 | 0·0106 |
| 7 | ♀ | 143 „ | 183 | 20·8 | −0·446 | 0·0248 |
| 3 | ♀ | > 1 year | 263 | 17·6 | −0·484 | 0·0231 |
| 2 | ♂ | > 1 year | 336 | 0·7 | −0·443 | 0·0184 |
| 5 | ♂ | 427 days | 357 | 39·1 | −0·417 | 0·0215 |
| 4 | ♂ | 442 days | 376 | 23·7 | −0·403 | 0·0253 |

* S.D. (standard deviation) $\times \sqrt{\frac{n}{n-1}}$ where $n$ = number of animals in group.

According to Hatai (1918) sex differences were apparent in the refractive index (RI) values of rat serum. He found that male rats older than 85 days had, on the average, higher serum RI values than females of this age group. This does not accord with trends observed in the present work and the explanation for the discrepancy is not clear, since both OR and RI might be expected to change in the same direction with changes in serum protein levels.

### *Serum optical rotation as a function of the total body weight (TBW) of normal rats*

Since the ages of some normal rats were not known with certainty the relationship between SOR (one determination per rat) and TBW was investigated. These values are plotted in the scatter diagram (Fig. 1) for 66 normal rats (32 ♂ and 34 ♀). Of these animals, 24 (13 ♂ and 11 ♀) belonged to the age group 70–120 days.

selected for comparison with the cancer group. Chest blood serum was examined in this group. For the remaining 42 animals tail blood serum was investigated.

Correlation analyses were made in the following categories :

(*a*) The cancer control group. For convenience, the negative sign of the rotation has been discarded, the value of α multiplied by 100 and the SOR value quoted as 100α. The following means and standard deviations were calculated :

$$\overline{TBW} = 192{\cdot}625000 \qquad S.D. = 23{\cdot}653422$$

$$\overline{100\alpha} = 40{\cdot}083333 \qquad S.D. = 1{\cdot}901468$$

$$\overline{TBW \times 100\alpha} = 7713{\cdot}09166$$

which gave the small negative correlation coefficient, $r = -0{\cdot}177$. In the t test

$$\left(t = \frac{r\sqrt{N-2}}{\sqrt{1-r^2}}, \text{ where } N = 24\right)$$

$t$ is 0·843, a value which falls below the 5 per cent probability level. Hence the correlation is not significant as was to be expected from the homogeneous nature

FIG. 1.—Scatter diagram showing relationship between rat total body weight and serum optical rotation (100 α). ● male rats ; ○ female rats ; ♀ rats in the cancer control group. The regression lines were plotted from the equations given in Table II for Group Ia ($r = +0{\cdot}495$)

of the sample with respect to age. Further information about this group is contained in the histogram (Fig. 3).

(*b*) All normal rats. Correlation analyses of TBW and SOR were made. Since the trend of the points in Fig. 1 indicates curvilinear rather than linear relationships, correlations were also made of the logarithms (to base 10) of TBW and 100α. In view of the marked tendency for female rat SOR values to exceed male rat values the sexes were analysed separately as above. Correlation coefficients together with tests of significance and regression equations are given in Table II.

Despite the sex difference trend seen in Fig. 1 the difference between the mean SOR values for male and female groups was less than twice the standard error of the difference of the means and was therefore not significant. However, the difference in the mean TBW'S for the groups was four times the standard error of the difference of the means. It is thought that the uneven weight distribution in the sexes obscures the sex difference in SOR.

The significance of the difference between the correlation coefficients (Table II) for male and female groups was tested by the $z$ transformation (Chambers, 1952) and it was found that the difference between the $z$'s was not significant.

TABLE II.—*Correlation Analyses of the Effect of Rat Total Body Weight (TBW) on Serum Optical Rotation* (100α).

Group I : Correlations between TBW ($x$) and 100 α ($y$) for ($a$) 66 ♂ and ♀ rats (b) 32 ♂ rats (c) 34 ♀ rats.

Group II : Correlations between log TBW ($x$) and log 100 α ($y$) for (a) 66 ♂ and ♀ rats (b) 32 ♂ rats (c) 34 ♀ rats.

| Group. | | r. | t.* | Regression equations. | Standard Errors of Estimate† for $x$ | $y$ |
|---|---|---|---|---|---|---|
| I | a | +0·495 | 4·558 | $x = 9·507\ y - 180·319$ <br> $y = 0·026\ x + 35·394$ | 58·365 | 3·039 |
| | b | +0·783 | 6·895 | $x = 22·480\ y - 660·888$ <br> $y = 0·027\ x + 33·483$ | 45·574 | 1·587 |
| | c | +0·718 | 5·835 | $x = 7·917\ y - 149·977$ <br> $y = 0·065\ x + 29·850$ | 31·214 | 2·831 |
| II | a | +0·592 | 5·873 | $x = 2·1866\ y - 1·2227$ <br> $y = 0·1601\ x + 1·2409$ | 0·1108 | 0·0300 |
| | b | +0·787 | 6·986 | $x = 3·7722\ y - 3·6835$ <br> $y = 0·1642\ x + 1·2141$ | 0·0839 | 0·0175 |
| | c | +0·809 | 7·790 | $x = 2·1240\ y - 1·1944$ <br> $y = 0·3083\ x + 0·9259$ | 0·0658 | 0·0251 |

* Each value exceeds 0·1 per cent probability level ; highly significant.
† Standard Error of Estimate = $S.D._{x \text{ or } y} \times \sqrt{1 - r^2}$

*Decline in serum optical rotation as a function of tumour percentage.*

Fig. 2 shows the relationship between SOR and tumour percentage for 45 animals. From the following data :

$$\overline{T\%} = 6·070200 \qquad S.D. = 5·381959$$

$$\overline{100\alpha} = 32·522222 \qquad S.D. = 4·245464$$

$$\overline{T\% \times 100\alpha} = 177·073109$$

$r$ was calculated to be — 0·890 with $t = 12·823$ (exceeds 0·1 per cent probability level ; highly significant). Regression equations (where $x$ = T per cent and $y = 100\alpha$) are as follows :

$$y = -0·702328x + 36·785493$$
$$x = -1·128679y + 42·777349$$

the standard errors of estimate for $x$ and $y$ being 2·450395 and 1·932951 respectively.

On correlating tumour weight with 100α a smaller negative coefficient, namely, $r = -0·84$ was obtained. Here $t$ was 10·173 (exceeds 0·1 per cent probability level) and the result is highly significant.

When total body weight of tumour animals was correlated with 100α, a low positive coefficient, $r = +0·018$ was found ($t = 0·121$) which was not significant.

In contrast, it may be of interest to mention that from figures quoted by El Mehairy (1950) for TBW of 33 tumour mice and their blood α-amino nitrogen levels, calculation has shown a positive correlation coefficient of 0·518 ($t$ = 3·371, significant at the 1 per cent level). Perhaps part of the increase in α-amino nitrogen observed during tumour growth (T per cent compared with α-amino $N$ gave $r = + 0{\cdot}753$) may be attributed to the TBW of the host. The result suggests that there may be a positive correlation between blood α-amino nitrogen and the TBW of normal mice but, unfortunately, this could not be checked for lack of data.

FIG. 2.—Scatter diagram showing relationship between serum optical rotation (100 α) and tumour percentage. ● male rats ; ○ female rats. Mean SOR (→→) and range (→) for 24 rats of control group. For regression equations see text.

From the histograms of Fig. 3 it will be noted that of 8 tumours whose SOR values overlap the normal histogram only one is greater than 1 per cent. The others are less than 0·5 per cent. Outside the normal range are 7 tumours smaller than 1 per cent. and of these 3 are less than 0·5 per cent.

*Tail bleeding experiments with normal, pregnant and tumour rats*

Examination of sera collected from normal adult female rats at intervals of not less than 4 days revealed that considerable fluctuations in SOR can occur. However the lowest values in any series have always been within the normal range for adult rats. Perhaps changes in total blood volume are responsible for the variations.

During pregnancy SOR tends to rise, drops sharply just before parturition and thereafter returns to normal. In the following example SOR and TBW (g.) at the time of tail bleeding are given in brackets.

20.ix.55 (— 0·409° ; 265) ; 22.ix.55 (mated) ; 27.ix.55 (— 0·411° ; 278) ; 3.x.55 (— 0·460° ; 272) ; 7.x.55 (— 0·474° ; 293) ; 11.x.55 (— 0·383° ; 335 ; lipaemic serum) ; 13.x.55 (parturition) ; 18.x.55 (— 0·436° ; 283).

Despite the considerable drop in SOR just before parturition, the final SOR value was within the normal range. In another pregnancy showing very similar trends

FIG. 3.—Histograms showing the distribution of serum optical rotations (100 $\alpha$) for cancer and normal rats. Each square represents one animal. The upper and lower figure in each square gives the T% and age of transplant (days) respectively.

| Kind and number of rats | $n$ | Mean 100$\alpha$. | S.D. | Difference of means. | S.E.† |
|---|---|---|---|---|---|
| Tumour (T) | 45 | 32·522222 | 4·245464 | 7·561111 | 0·74 |
| Normal (N) | 24 | 40·083333 | 1·901468 | | |

Difference significant.

the lowest SOR value encountered was — 0·393° one day after parturition. In another case a pregnant animal was chest bled just before parturition. The SOR was — 0·381° and the foetal weight was 37 g. (or 11 per cent of TBW). Again, two animals chest bled during early pregnancy gave normal SOR values, namely, — 0·447° and — 0·436° and the foetal percentages were 2·3 and 1·0 respectively.

During tumour growth the drop in SOR begins usually in the second week following transplantation, when acceleration of neoplastic growth sets in. The following is a typical experiment. When two values for SOR are quoted the second refers to a measurement on chest blood serum. TBW is given in brackets.

| Male Rat Number. | 23.vii.55 | 25.vii.55 | 1.viii.55 | 6.viii.55 | 8.viii.55 | T%. |
|---|---|---|---|---|---|---|
| 1 | −0·407 (196) | Rd/3 inoculated | −0·415 (207) | −0·397 (208)<br>−0·374 | | — |
| 2 | −0·411 (222) | | −0·395 (235) | −0·363 (243)<br>−0·344 | | 3·6 |
| 3 | −0·414 (215) | | −0·403 (208) | | −0·293 (224)<br>−0·282 | 9·6 |

*Salmonella enteritidis infections of some Rd/3 tumours*

Reference has already been made to the appearance of *S. enteritidis* in some Rd/3 tumours (Neish, 1955)* and, in the present work, several tumours which were growing slowly or regressing were found to contain this organism. One of the " tumours " (0·5 per cent) appeared to consist entirely of pus from which only *S. enteritidis* could be isolated. The host gave a normal SOR value (— 0·405°). In another tumour (0·7 per cent), which consisted of a cluster of nodules, only one of the nodules contained the organism and the host's SOR was abnormal (—0·364°). Possibly some of the rats are carriers of silent *S. enteritidis* which becomes activated after Rd/3 inoculation. It would appear, however, that apart from the apparent adverse effect of *S. enteritidis* on tumour growth, the presence of the organism has little influence on SOR.

It is of interest that two human cancer cases have been recorded with salmonella abscesses in the tumours, one by Gray (1936 ; *S. suipestifer* in myoma) and the other by Giel (1954 ; *S. typhimurium* in pheochromocytoma).

### DISCUSSION

Rd/3 tumours as small as 0·5 to 1 per cent can cause a marked diminution in the host's serum optical rotation. As an indicator of tumour growth the SOR procedure seems to be more sensitive than e.g. Warburg and Christian's (1943) plasma aldolase study in which abnormally high aldolase levels appeared only when the Jensen sarcoma occupied 2·5 per cent of the rats' total body weight. Again in the study of El Mehairy (1950) increases in mouse blood α-amino nitrogen, as the result of tumour growth, did not reach significantly high levels until the tumours were of the order of 5 per cent of the total body weight.

The results obtained with normal animals in the present work seem to indicate that when tests are being applied to serum proteins (and perhaps even to amino

* When this communication was made the writer was unaware of the paper by Schwartzman (Schwartzman, G., 1935, *Proc. Soc. exp. Biol. N.Y.*, **32**, 1603) in which it was shown that spontaneous *B. enteritidis* infection of mice bearing Sarcoma 180 caused regression of many of the tumours.

acids, proteoses and other materials in equilibrium with the proteins) effects due to the age of the serum donor may have to be taken into account.

While the magnitude of the drop in SOR during pregnancy may be as great as that observed during tumour growth the SOR level increases to such an extent before parturition that the lowest SOR level finally attained is still within the normal range. No such elevation in SOR has ever been noted during tumour development.

Although it is probable that hypoalbuminaemia (cf. Winzler, 1953) may be the main factor for the drop in SOR during tumour growth, the possibility of contributions from " pathological " proteins with lower optical rotations than normal (Jirgensons, 1955) cannot be excluded. Changes in blood volume may also play some part. With these points in mind a more detailed investigation is being undertaken.

### SUMMARY

The optical rotation of rat serum has been shown to be dependent on the age (and weight) of the animal.

During the development of Rd/3 tumour, the optical rotation of the host's serum declines markedly.

I am greatly indebted to Dr. R. R. Gillies of the Dept. of Bacteriology for his identification of *S. enteritidis* in the tumour pus samples and I wish to thank Mr. A. Purdie for his great care in supplying and maintaining the animals.

Thanks are due to the Melville Trust for a fellowship in cancer research.

### REFERENCES

ABDERHALDEN, E. AND WEIL, A.—(1912) *Z. physiol. Chem.*, **81**, 233.

CHAMBERS, E. G.—(1952) 'Statistical Calculation for Beginners.' Cambridge (University Press), 2nd edition, p. 63.

EL MEHAIRY, M. M.—(1950) *Brit. J. Cancer*, **4**, 195.

GIEL, C. P.—(1954) *New Engl. J. Med.*, **251**, 980.

GRAY, L. A.—(1936) *Johns Hopk. Hosp. Bull.*, **59**, 231.

HATAI, S.—(1918) *J. biol. Chem.*, **35**, 527.

JIRGENSONS, B.—(1955) *Cancer*, **8**, 809.

NEISH, W. J. P.—(1954*a*) *Brit. J. Cancer*, **8**, 361.—(1954*b*) *Ibid.*, **8**, 566.—(1955) *Naturwissenschaften*, **42**, 420.

SWANSON,P. P. AND SMITH, A. H.—(1932) *J. biol. Chem.*, **97**, 745.

WARBURG, O. AND CHRISTIAN, W.—(1943) *Biochem. Z.*, **314**, 399.

WINZLER, R. J.—(1953) *Plasma Proteins in Cancer in Advances in Cancer Research*, **1**, 503.